# Hay Fever*

**Published:** 1887-09

**Authors:** J. A. Biegler

**Affiliations:** Rochester, N. Y.


### HAY FEVER.*

J. A. Biegler, M. D., Rochester, N. Y.

Case.—Professor N. W. B., for many years a sufferer from this disease in its severe form. As is usual with that class of sufferers, he could obtain partial relief by a sojourn in favorable regions, but his duties of teacher brought him home yearly in the midst of his sickness, when he was conspicuous in the streets on account of his lachrymose trouble.

September 24th, 1886, at the time when his suffering was at its highest, at eight P. M. I gave him one dose of Lycopod.$^{cm}$, which gave him permanent relief within twenty-four hours.

The most prominent and troublesome symptom, of which he had for several days complained, and which mostly determined the choice of the remedy, was a very troublesome ITCHING IN THE CANTHI, and which he begged relief for more than for the rest of his sufferings. The remedy was not selected on this one indication, but because it also covered, more than any other, the

* This paper was read by Dr. Biegler at the last meeting of the I. H. A. Both paper and the discussion which followed are so instructive that we give them in full.—Eds. H. P.

symptoms of the nose and eyes, and which are to be found in the proving as given in the *Chronic Diseases* and Allen's *Encyclopœdia,* to which I refer for the leading symptoms here given.

